# Supplementary material for: The Evolution and Characterization of the RNA Interference Pathways in Lophotrochozoa
Source: Genome Biol Evol. 2024 May 7;16(5):evae098. doi: 10.1093/gbe/evae098 (PMC11114477; doi:10.1093/gbe/evae098)
Supplement: evae098_Supplementary_Data [file evae098_supplementary_data.zip › Supplementary_materials.zip!/selected_miRNAs/NoGe_selected_miRNAs/mirdeep_pdf_files/NMRB01000024.1_Notospermus_geniculatus_strain_Ushimado_scaffold24,_whole_genome_shotgun_sequence_1011.pdf]

Provisional ID	:	NMRB01000024.1_Notospermus_geniculatus_strain_Ushimado_scaffold24,_whole_genome_shotgun_sequence_1011
Score total	:	19674.7
Score for star read(s)	:	3.9
Score for read counts	:	19666.1
Score for mfe	:	1.7
Score for randfold	:	0
Score for cons. seed	:	3
Total read count	:	38586
Mature read count	:	38450
Loop read count	:	0
Star read count	:	136

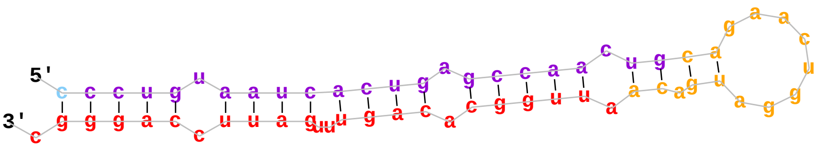

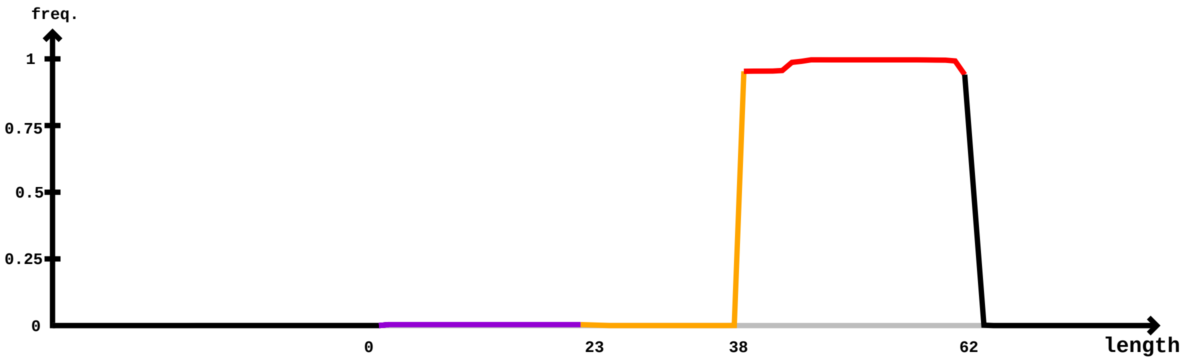

Star Mature

```
5'- gaugauggaacaaccacagucgugguuuccgccccuguaaucacugagccaacugcagaacuggaugacaauuggcacaguuugauuccagggcugaaauccacaucaaccua  -3'  obs
    gaugauggaacaaccacagucgugguuuccgcccuguaaucacugagccaacugcagaacuggaugacaauuggcacaguuugauuccagggcugaaauccacaucaaccua        exp
    ((((.((((...(((((....)))))(((.((((.((.((((((((.(((((.((((........)).)).))))).)))).)))).)))))).))).))))))))).....   reads     mm      sample
    ............ccacagucgugguuuccgcc.................................................................................   1         0       Fm3
    ............ccacagucgugguuucAgcc.................................................................................   1         1       Fm3
    .................................ccuguaaucacugagccaacu...........................................................   1         0       Fm3
    .................................ccuguaaucacugagccaacug..........................................................   17        0       Fm3
    ......................................................................auuggcacaguuugauucc........................   8         0       Fm3
    ......................................................................auuggcacaguuugauuccag......................   1         0       Fm3
    ......................................................................auuggcacaguuugauuUcagg.....................   1         1       Fm3
    ......................................................................auuggcacaguuugauuccagg.....................   24        0       Fm3
    ......................................................................auuggcacaguuugauuccaggU....................   1         1       Fm3
    ......................................................................auuggcacUguuugauuccaggg....................   1         1       Fm3
    ......................................................................auuggcacaguuugauuccaggg....................   234       0       Fm3
    ......................................................................auuggcacaguuugauuUcaggg....................   1         1       Fm3
    ......................................................................auuggcacaguuugauuAcaggg....................   1         1       Fm3
    ......................................................................auuggcacaguuugauuGcaggg....................   1         1       Fm3
    ......................................................................auuggcacaguuugaCuccaggg....................   1         1       Fm3
    ......................................................................auuggcacagGuugauuccaggg....................   1         1       Fm3
    ......................................................................auuggcacaguuugauGccaggg....................   1         1       Fm3
    ......................................................................auugCcacaguuugauuccagggc...................   1         1       Fm3
    ......................................................................auuggcaAaguuugauuccagggc...................   1         1       Fm3
    ......................................................................aGuggcacaguuugauuccagggc...................   1         1       Fm3
    ......................................................................auuAgcacaguuugauuccagggc...................   1         1       Fm3
    ......................................................................Uuuggcacaguuugauuccagggc...................   1         1       Fm3
    ......................................................................auuCgcacaguuugauuccagggc...................   1         1       Fm3
    ......................................................................auuggcacaguuugauuUcagggc...................   14        1       Fm3
    ......................................................................auuggcacaguCugauuccagggc...................   2         1       Fm3
    ......................................................................auuggcacagAuugauuccagggc...................   2         1       Fm3
    ......................................................................aAuggcacaguuugauuccagggc...................   1         1       Fm3
    ......................................................................auuggcacaguuugauuccagggA...................   38        1       Fm3
    ......................................................................auuggcacaguuugauuccaggAc...................   2         1       Fm3
    ......................................................................auuggcacaguuugauuccaAggc...................   1         1       Fm3
    ......................................................................auuggcNcaguuugauuccagggc...................   1         1       Fm3
    ......................................................................auuggcacaguuugauuAcagggc...................   23        1       Fm3
    ......................................................................auuggcacaguuugauuccagggc...................   2070      0       Fm3
```

Star Mature

```
gaugauggaacaaccacagucgugguuuccgccccuguaaucacugagccaacugcagaacuggaugacaauuggcacaguuugauuccagggcugaaauccacaucaaccua
......................................................................auuggcacCguuugauuccagggc...................   1     1   Fm3
......................................................................auuggcacagCuugauuccagggc...................   1     1   Fm3
......................................................................auuggcacaguuugauucUagggc...................   2     1   Fm3
......................................................................auuggAacaguuugauuccagggc...................   1     1   Fm3
......................................................................auuggcacaguuugauuccaUggc...................   1     1   Fm3
......................................................................auuggcacaguuGgauuccagggc...................   1     1   Fm3
......................................................................auuggcacaguuugauucAagggc...................   1     1   Fm3
......................................................................auuggcacUguuugauuccagggc...................   2     1   Fm3
......................................................................auuggcacaguuugUuuccagggc...................   1     1   Fm3
......................................................................auuggcacaguuugauCccagggc...................   7     1   Fm3
......................................................................auuggcacaguuuCauuccagggc...................   1     1   Fm3
......................................................................auCggcacaguuugauuccagggc...................   1     1   Fm3
......................................................................auuggcacaguuugauuccagggU...................   59    1   Fm3
......................................................................auuggcacaguuugauuccaggUc...................   1     1   Fm3
......................................................................auuggcaUaguuugauuccagggc...................   1     1   Fm3
......................................................................auuggcacaguuugauAccagggc...................   8     1   Fm3
......................................................................auuggcacaguuugauGccagggc...................   2     1   Fm3
......................................................................auuggcacaUuuugauuccagggc...................   1     1   Fm3
......................................................................auuUgcacaguuugauuccagggc...................   2     1   Fm3
......................................................................auuggcacaguuugauuGcagggc...................   6     1   Fm3
......................................................................auuggcacagGuugauuccagggc...................   2     1   Fm3
......................................................................auuggcacaguuugauucGagggc...................   1     1   Fm3
......................................................................auuggcacaguuugauuccagggcu..................   34    0   Fm3
......................................................................auuggUacaguuugauuccagggcu..................   1     1   Fm3
......................................................................auuggcacaguuugauuccagggUu..................   2     1   Fm3
......................................................................auuggcacaguuugauuccagggcA..................   2     1   Fm3
......................................................................auuggcacaguuugauuccagggcuA.................   1     1   Fm3
......................................................................auuggcacaguuugauuccagggcug.................   1     0   Fm3
.......................................................................uuggcacaguuugauuccagggc...................   3     0   Fm3
...........................................................................cacaguuugauuccaggg....................   2     0   Fm3
...........................................................................cacaguuugauuccaggCc...................   2     1   Fm3
...........................................................................cacaguuugauuccaggAc...................   1     1   Fm3
...........................................................................cacaguuugauuccagCgc...................   1     1   Fm3
...........................................................................cacaguuugauucAagggc...................   1     1   Fm3
...........................................................................cacaguuugauuccagggc...................   89    0   Fm3
...........................................................................cacaguuugauuccagAgc...................   1     1   Fm3
...........................................................................cacaguuugauuccaggUc...................   2     1   Fm3
...........................................................................cacaguuugaGuccagggc...................   1     1   Fm3
...........................................................................cacaguuugauuccagggcu..................   7     0   Fm3
............................................................................acaguuugauuccagggc...................   7     0   Fm3
.............................................................................caguuugauuccagggcC..................   1     1   Fm3
.............................................................................caguuugauuccagggcu..................   23    0   Fm3

.................................ccuguaaucacugagccaacu...........................................................   1     0   Mm3
.................................ccuguaaucacugagccCacug..........................................................   1     1   Mm3
.................................ccuguaaucacugagccGacug..........................................................   1     1   Mm3
.................................ccuguGaucacugagccaacug..........................................................   1     1   Mm3
.................................ccuguaaucaAugagccaacug..........................................................   1     1   Mm3
.................................ccuguaaucacugagccaacug..........................................................   20    0   Mm3
.................................ccuguaaucacugagccaacugcagaacu...................................................   1     0   Mm3
.................................ccuguaaucacugagccaacugcagaacuggaugaca...........................................   1     0   Mm3
......................................................................auuggcacaguuugauuc.........................   1     0   Mm3
......................................................................auuggcacaguuugauuccag......................   1     0   Mm3
......................................................................auuggcacaguuugauuccagg.....................   15    0   Mm3
......................................................................auAggcacaguuugauuccagg.....................   1     1   Mm3
......................................................................auuggcacagAuugauuccagg.....................   1     1   Mm3
......................................................................auuggcacaguuugauuccaggA....................   2     1   Mm3
......................................................................auCggcacaguuugauuccaggg....................   2     1   Mm3
......................................................................auuggcacaguuugauuccaggg....................   220   0   Mm3
......................................................................auuggcacaguuugauuUcaggg....................   4     1   Mm3
......................................................................auuggcacaguuugauCccaggg....................   1     1   Mm3
......................................................................auuggcacagAuugauuccaggg....................   1     1   Mm3
......................................................................auuggcacaguuugauuccaggU....................   2     1   Mm3
......................................................................auuggcacaguuugauuccagggc...................   1266  0   Mm3
......................................................................auuggcacagAuugauuccagggc...................   2     1   Mm3
......................................................................auuggcacaguuugauuccagCgc...................   1     1   Mm3
......................................................................auuggcacaguuugauuccUgggc...................   1     1   Mm3
......................................................................auuggcacaguuugauCccagggc...................   1     1   Mm3
......................................................................auuAgcacaguuugauuccagggc...................   1     1   Mm3
......................................................................Cuuggcacaguuugauuccagggc...................   2     1   Mm3
```

```
                                 Star                                 Mature
gaugauggaacaaccacagucgugguuuccgccccuguaaucacugagccaacugcagaacuggaugacaauuggcacaguuugauuccagggcugaaauccacaucaaccua
......................................................................auuggcacaguGugauuccagggc...................    1     1    Mm3
......................................................................auuggcacaguuugauucUagggc...................    2     1    Mm3
......................................................................auuggcacaguuuAauuccagggc...................    1     1    Mm3
......................................................................Guuggcacaguuugauuccagggc...................    1     1    Mm3
......................................................................auuggcGcaguuugauuccagggc...................    5     1    Mm3
......................................................................auuggcacagGuugauuccagggc...................    2     1    Mm3
......................................................................auuggcacaguuugauuGcagggc...................    3     1    Mm3
......................................................................auuggcacaguuugauuAcagggc...................    16    1    Mm3
......................................................................Nuuggcacaguuugauuccagggc...................    1     1    Mm3
......................................................................auuggcacaguuugauGccagggc...................    7     1    Mm3
......................................................................auuggcacagCuugauuccagggc...................    2     1    Mm3
......................................................................auuggcacGguuugauuccagggc...................    1     1    Mm3
......................................................................auuggcacaguuugauuccagggU...................    24    1    Mm3
......................................................................auuggcacaguuugauuccagggA...................    12    1    Mm3
......................................................................auuggcacaguuugauuUcagggc...................    5     1    Mm3
......................................................................auuggcacUguuugauuccagggc...................    1     1    Mm3
......................................................................auuggcacaguuugauuccagggUu..................    1     1    Mm3
......................................................................auuggcacaguuugauuAcagggcu..................    3     1    Mm3
......................................................................auuggcacGguuugauuccagggcu..................    1     1    Mm3
......................................................................auuggcacaguuugauuccagggcA..................    3     1    Mm3
......................................................................auuggcacaguuugauuccagggcu..................    65    0    Mm3
......................................................................auuggcacaguuugauuccagggAu..................    1     1    Mm3
......................................................................auuggcacaguuugauuccagggcuU.................    5     1    Mm3
......................................................................auuggcacaguuugauuccagggcug.................    1     0    Mm3
........................................................................uggcacaguuugauuccagggc...................    1     0    Mm3
..........................................................................gcacaguuugauuccaggg....................    1     0    Mm3
..........................................................................gcacaguuugauuccaggUc...................    1     1    Mm3
..........................................................................gcaAaguuugauuccagggc...................    1     1    Mm3
..........................................................................gcacaguuugauuccagUgc...................    1     1    Mm3
..........................................................................gcacaguuugauuccagggc...................    28    0    Mm3
..........................................................................gcacaguuugauuccagggcu..................    1     0    Mm3
...........................................................................cacaguuugauuccagggU...................    1     1    Mm3
...........................................................................cacaguuugauuccagggc...................    31    0    Mm3
...........................................................................cacaguuugauuccagggcA..................    2     1    Mm3
...........................................................................cacaguuugauuccagggcu..................    4     0    Mm3
...........................................................................cacaguuugauuccagggcuU.................    3     1    Mm3
............................................................................acaguuugauuccagggc...................    4     0    Mm3
............................................................................acaguuugauuccaggUc...................    1     1    Mm3
............................................................................acaguuugauAccagggc...................    1     1    Mm3
............................................................................acaguuugauuccagggcu..................    2     0    Mm3
............................................................................acaguuugauuccagggcA..................    1     1    Mm3
.............................................................................caguuugauuccagggcA..................    1     1    Mm3
.............................................................................caguuugauuccagggcu..................    17    0    Mm3
.............................................................................caguuugauuccagggcuU.................    3     1    Mm3

......................................................................auuggcacaguuugauucca.......................    11    0    Fm2
......................................................................auuggcacaguuugauuAca.......................    1     1    Fm2
......................................................................auuggcacaguuugauuccag......................    4     0    Fm2
......................................................................auuggcacaguuugauuccagg.....................    11    0    Fm2
......................................................................auuggcacaguuugauuGcaggg....................    2     1    Fm2
......................................................................auuggcacUguuugauuccaggg....................    1     1    Fm2
......................................................................auuggcacaguuugauuccaggU....................    5     1    Fm2
......................................................................auuggcacaguuugauuccaggg....................    129   0    Fm2
......................................................................auuggcacaguuugauuAcaggg....................    1     1    Fm2
......................................................................auuggcacGguuugauuccagggc...................    3     1    Fm2
......................................................................auuggcacaguuugauuccGgggc...................    3     1    Fm2
......................................................................auuggUacaguuugauuccagggc...................    1     1    Fm2
......................................................................auGggcacaguuugauuccagggc...................    2     1    Fm2
......................................................................auuggcacaguuugauuccagggc...................    4659  0    Fm2
......................................................................auugUcacaguuugauuccagggc...................    1     1    Fm2
......................................................................auuggcacaguuugauuGcagggc...................    28    1    Fm2
......................................................................auuggcacaguuugauuccagAgc...................    2     1    Fm2
......................................................................auuUgcacaguuugauuccagggc...................    2     1    Fm2
......................................................................auuggcacaguuugauuccaggAc...................    1     1    Fm2
......................................................................auuggcacaguuugauuccagggU...................    14    1    Fm2
......................................................................auuCgcacaguuugauuccagggc...................    1     1    Fm2
......................................................................auuggcacagGuugauuccagggc...................    5     1    Fm2
......................................................................auuggcacaguuugauuUcagggc...................    41    1    Fm2
......................................................................auuggcaUaguuugauuccagggc...................    3     1    Fm2
......................................................................auuggcacaguuugauuAcagggc...................    55    1    Fm2
```

Star Mature

gaugauggaacaaccacagucgugguuuccgccccuguaaucacugagccaacugcagaacuggaugacaauuggcacaguuugauuccagggcugaaauccacaucaaccua			
....auuggcacagAuugauuccagggc...................	6	1	Fm2
....auuggcacaguuugauuccagCgc...................	1	1	Fm2
....auuggcacUguuugauuccagggc...................	3	1	Fm2
....auCggcacaguuugauuccagggc...................	1	1	Fm2
....Nuuggcacaguuugauuccagggc...................	2	1	Fm2
....auuggcacaguuugauAccagggc...................	9	1	Fm2
....auuggcacaUuuugauuccagggc...................	2	1	Fm2
....auuggcacaguuugauuccaUggc...................	2	1	Fm2
....auuggcacagCuugauuccagggc...................	3	1	Fm2
....auuggNacaguuugauuccagggc...................	1	1	Fm2
....aGuggcacaguuugauuccagggc...................	1	1	Fm2
....auuggcacaguuugauuccagggA...................	19	1	Fm2
....Uuuggcacaguuugauuccagggc...................	3	1	Fm2
....Guuggcacaguuugauuccagggc...................	3	1	Fm2
....auuggcacaguuugauCccagggc...................	13	1	Fm2
....auuggcUcaguuugauuccagggc...................	2	1	Fm2
....auuggcacaguuugauuccagUgc...................	2	1	Fm2
....auuAgcacaguuugauuccagggc...................	1	1	Fm2
....auuggcacaguuugauGccagggc...................	15	1	Fm2
....auuggcacaguGugauuccagggc...................	1	1	Fm2
....auuggcacaguuugauuccaggUc...................	1	1	Fm2
....auuggcacaguuugauuccagggcA..................	18	1	Fm2
....auuggcacaguuugauuccagggcu..................	55	0	Fm2
....auuggcacaguuugauAccagggcu..................	1	1	Fm2
....auuggcacaguuugauuccagggcG..................	4	1	Fm2
....auuggcacaguuugaCuccagggcu..................	1	1	Fm2
....auuggcacaguuugauuUcagggcu..................	1	1	Fm2
....auuggcacagGuugauuccagggcu..................	1	1	Fm2
....auuggcacaguuugauuGcagggcu..................	1	1	Fm2
....uuggcacaguuugauuccagggc...................	5	0	Fm2
....cacaguuugauuccagggc...................	95	0	Fm2
....cacaguuugaAuccagggc...................	1	1	Fm2
....cacaguuugauuccaggCc...................	1	1	Fm2
....cacaguuugauuccagggcG..................	2	1	Fm2
....cacaguuugauuccagggcu..................	4	0	Fm2
....acaguuugauuccagggc...................	7	0	Fm2
....acaguuugauuccagggcu..................	2	0	Fm2
....caguuugauuccagggcu..................	17	0	Fm2
....caguuugauuccagggcG..................	2	1	Fm2
.................................ccuguaaucacugagccaacug..	15	0	Fm1
.................................ccuguaaucacugagccaacugc...	2	0	Fm1
....Cauuggcacaguuugauuccaggg....................	3	1	Fm1
....auuggcacaguuugauucc........................	2	0	Fm1
....auuggcacaguuugauucca.......................	6	0	Fm1
....auuggcacaguuugauuccaU.......................	1	1	Fm1
....auuggcacaguuugauuccagg.....................	19	0	Fm1
....auuggcacaguuugauGccagg.....................	1	1	Fm1
....auuggcacaguuugauuccaggU....................	9	1	Fm1
....auuggcacaguuugauuUcaggg....................	4	1	Fm1
....auuggcacaguuugauuccCggg....................	1	1	Fm1
....auuggcacagAuugauuccaggg....................	1	1	Fm1
....auuggcacaguuugauuccaggg....................	395	0	Fm1
....auuggcacaguuugauuGcaggg....................	1	1	Fm1
....auuggcacaguuugauuccGggg....................	1	1	Fm1
....auuAgcacaguuugauuccaggg....................	1	1	Fm1
....auuggcacaguuugauuAcaggg....................	2	1	Fm1
....auuggcacaguuugauAccaggg....................	3	1	Fm1
....auuggcacaguuugauGccaggg....................	1	1	Fm1
....auuggcacaguuugauCccagggc...................	26	1	Fm1
....auuggcacaguuugauuAcagggc...................	66	1	Fm1
....auuggcacaguuAgauuccagggc...................	1	1	Fm1
....auuggcacaguuugauuccaggUc...................	2	1	Fm1
....aAuggcacaguuugauuccagggc...................	2	1	Fm1
....auuggcacagCuugauuccagggc...................	9	1	Fm1
....auuggcacaAuuugauuccagggc...................	1	1	Fm1
....auuggcacaguuugauuccagggA...................	16	1	Fm1
....auuggcaUaguuugauuccagggc...................	2	1	Fm1
....auuggcacaguuuUauuccagggc...................	1	1	Fm1
....auugCcacaguuugauuccagggc...................	1	1	Fm1

```
                                 Star                             Mature
gaugauggaacaaccacagucgugguuuccgccccuguaaucacugagccaacugcagaacuggaugacaauuggcacaguuugauuccagggcugaaauccacaucaaccua
......................................................................auuggcacaguuugauuUcagggc................... 37 1 Fm1
......................................................................auuggcacaguuugauuccagggG................... 1 1 Fm1
......................................................................auuggcacaguuugauucAagggc................... 1 1 Fm1
......................................................................auuggcacaguuugauuccaggAc................... 3 1 Fm1
......................................................................auuggcacaguuugUuuccagggc................... 1 1 Fm1
......................................................................auuggcacaguuugauuGcagggc................... 29 1 Fm1
......................................................................auuggUacaguuugauuccagggc................... 2 1 Fm1
......................................................................auuggcacagGuugauuccagggc................... 9 1 Fm1
......................................................................Nuuggcacaguuugauuccagggc................... 2 1 Fm1
......................................................................auuggcacaguuugauAccagggc................... 17 1 Fm1
......................................................................Guuggcacaguuugauuccagggc................... 8 1 Fm1
......................................................................auuggcacagAuugauuccagggc................... 9 1 Fm1
......................................................................auuggcacaUuuugauuccagggc................... 1 1 Fm1
......................................................................auuggcacaguuugauuccagUgc................... 1 1 Fm1
......................................................................auuggcaGaguuugauuccagggc................... 2 1 Fm1
......................................................................auugAcacaguuugauuccagggc................... 3 1 Fm1
......................................................................aCuggcacaguuugauuccagggc................... 1 1 Fm1
......................................................................auuggcacaguuugauuccCgggc................... 1 1 Fm1
......................................................................auuggcacaguuugauuccagggc................... 7782 0 Fm1
......................................................................auuggcacaguuugaCuccagggc................... 2 1 Fm1
......................................................................auugUcacaguuugauuccagggc................... 3 1 Fm1
......................................................................auuggcacaguuugauuccagAgc................... 2 1 Fm1
......................................................................Uuuggcacaguuugauuccagggc................... 6 1 Fm1
......................................................................auuggcacGguuugauuccagggc................... 2 1 Fm1
......................................................................auuggcacaguuugauGccagggc................... 19 1 Fm1
......................................................................auuggcGcaguuugauuccagggc................... 5 1 Fm1
......................................................................auuggcUcaguuugauuccagggc................... 4 1 Fm1
......................................................................auuggcacCguuugauuccagggc................... 1 1 Fm1
......................................................................auuggcacaguuugauuccGgggc................... 1 1 Fm1
......................................................................auuggcacaguuugGuuccagggc................... 1 1 Fm1
......................................................................auGggcacaguuugauuccagggc................... 4 1 Fm1
......................................................................auCggcacaguuugauuccagggc................... 2 1 Fm1
......................................................................auuggcacaguuugauuccagggU................... 91 1 Fm1
......................................................................auuggcacUguuugauuccagggc................... 13 1 Fm1
......................................................................auuggNacaguuugauuccagggc................... 1 1 Fm1
......................................................................auuUgcacaguuugauuccagggc................... 1 1 Fm1
......................................................................auuggcacaguuugauuccagggcA.................. 9 1 Fm1
......................................................................auuggcacaguuugauAccagggcu.................. 1 1 Fm1
......................................................................auuggcacaguuugauuccagggcG.................. 7 1 Fm1
......................................................................auuggcacagAuugauuccagggcu.................. 2 1 Fm1
......................................................................Cuuggcacaguuugauuccagggcu.................. 1 1 Fm1
......................................................................auuggcacaguuugauuccagggcu.................. 204 0 Fm1
......................................................................auuggcacaguuugauuUcagggcu.................. 2 1 Fm1
......................................................................auuggcacaguuugauuccagggUu.................. 1 1 Fm1
......................................................................auuggcacaguuugauucUagggcu.................. 1 1 Fm1
......................................................................auuggcacaguuugauuccagggcCg................. 1 1 Fm1
......................................................................auuggcacaguuugauuccagggcuU................. 6 1 Fm1
.......................................................................uuggcacaguuugauuccagggc................... 1 0 Fm1
..........................................................................gcacaguuugauuccagggc................... 4 0 Fm1
...........................................................................cacaguuugauuccaggA.................... 1 1 Fm1
...........................................................................cacaguuugauuccaggg.................... 5 0 Fm1
...........................................................................cacaguuugauuccaggUc................... 1 1 Fm1
...........................................................................cacaguuugauuccagggc................... 52 0 Fm1
...........................................................................cacaguuugauuccagggcu.................. 2 0 Fm1
............................................................................acaguuugauuccagggU................... 1 1 Fm1
............................................................................acaguuugauuccagggc................... 10 0 Fm1
............................................................................acaguuugauuccaggCc................... 1 1 Fm1
............................................................................acaguuugauuccagggcu.................. 3 0 Fm1
............................................................................acaguuugauuccagggcuU................. 2 1 Fm1
.............................................................................caguuugauuccagggcu.................. 19 0 Fm1
.............................................................................caguuugauuccagggcuU................. 4 1 Fm1

.................................ccuguaaucacugagccaacug.......................................................... 19 0 Mm1
......................................................................auuggcacaguuugauuccag...................... 3 0 Mm1
......................................................................auuggcacaguuugauuccagg..................... 21 0 Mm1
......................................................................auuggcacagCuugauuccagg..................... 1 1 Mm1
......................................................................auuggcacaguuugauuccaggg.................... 370 0 Mm1
......................................................................auuggcacagAuugauuccaggg.................... 1 1 Mm1
......................................................................auuggcacaguuugauuGcaggg.................... 4 1 Mm1
......................................................................auuggcacaUuuugauuccaggg.................... 1 1 Mm1
```

Star Mature

gaugauggaacaaccacagucgugguuuccgccccuguaaucacugagccaacugcagaacuggaugacaauuggcacaguuugauuccagggcugaaauccacaucaaccua			
....auuggcacaguuugauuAcaggg....................	2	1	Mm1
....auuggcacaguuugauuUcaggg....................	1	1	Mm1
....auGggcacaguuugauuccaggg....................	1	1	Mm1
....auuggcUcaguuugauuccaggg....................	1	1	Mm1
....auuggcacaguuugauAccaggg....................	3	1	Mm1
....auuggcacaguuugauuccaggA....................	5	1	Mm1
....auuggcaUaguuugauuccagggc...................	1	1	Mm1
....auuggcacaguuugCuuccagggc...................	2	1	Mm1
....auuCgcacaguuugauuccagggc...................	1	1	Mm1
....auuggcacaguuugauuccagggU...................	12	1	Mm1
....auuggcacaguuugauucGagggc...................	1	1	Mm1
....auAggcacaguuugauuccagggc...................	2	1	Mm1
....auuggcacaguuugauuGcagggc...................	23	1	Mm1
....auuggUacaguuugauuccagggc...................	1	1	Mm1
....auuggcacagAuugauuccagggc...................	13	1	Mm1
....auuggcacaguuugauucAagggc...................	1	1	Mm1
....auuggcacUguuugauuccagggc...................	8	1	Mm1
....auuggcGcaguuugauuccagggc...................	1	1	Mm1
....auuggcacaguuugauucUagggc...................	1	1	Mm1
....auuggcacaguuugauAccagggc...................	14	1	Mm1
....aAuggcacaguuugauuccagggc...................	5	1	Mm1
....auuggcacaguuugauuccaggAc...................	1	1	Mm1
....auuggcacaguuugauuAcagggc...................	49	1	Mm1
....Guuggcacaguuugauuccagggc...................	8	1	Mm1
....auuggcacaguuugauuccGggc...................	2	1	Mm1
....auuggcacaguuugauuccagAgc...................	1	1	Mm1
....auuggcacaguuugauGccagggc...................	17	1	Mm1
....auugUcacaguuugauuccagggc...................	1	1	Mm1
....auuggcacaguuugUuuccagggc...................	3	1	Mm1
....auuggcacaguuugauuccagggA...................	36	1	Mm1
....auuggcacagCuugauuccagggc...................	5	1	Mm1
....auuggcacaguuugGuuccagggc...................	1	1	Mm1
....auuggcacaguuugauuccagggc...................	5895	0	Mm1
....auuggcacaguuugaCuccagggc...................	2	1	Mm1
....aGuggcacaguuugauuccagggc...................	2	1	Mm1
....auuggcacaguAugauuccagggc...................	1	1	Mm1
....auuggcNcaguuugauuccagggc...................	1	1	Mm1
....auuggcacaguuugauCccagggc...................	20	1	Mm1
....auugCcacaguuugauuccagggc...................	1	1	Mm1
....auCggcacaguuugauuccagggc...................	5	1	Mm1
....auuggcacCguuugauuccagggc...................	1	1	Mm1
....Nuuggcacaguuugauuccagggc...................	2	1	Mm1
....auuggcacaguuugauuUcagggc...................	29	1	Mm1
....auuggcacagGuugauuccagggc...................	7	1	Mm1
....Uuuggcacaguuugauuccagggc...................	6	1	Mm1
....Cuuggcacaguuugauuccagggc...................	1	1	Mm1
....auuggcacaguuugauuUcagggcu..................	1	1	Mm1
....auuggcacaguuugauuccagggcu..................	74	0	Mm1
....auuggcacaguuugauuccagggcA..................	11	1	Mm1
....auuggcacaguuugauuccagggcug.................	2	0	Mm1
....auuggcacaguuugauuccagggcuU.................	4	1	Mm1
....uuggcacaguuugauuccaggg....................	1	0	Mm1
....uuggcacaguuugUuuccagggc...................	1	1	Mm1
....uuggcacaguuugauuccagggc...................	1	0	Mm1
....ggcacaguuugauuccagggc...................	3	0	Mm1
....Acacaguuugauuccagggc...................	1	1	Mm1
....gcacaguuugauuccagggc...................	26	0	Mm1
....gcacaguuugauuccagggcu..................	2	0	Mm1
....cacaguuugauuccaggg....................	2	0	Mm1
....Uacaguuugauuccagggc...................	1	1	Mm1
....cacaguGugauuccagggc...................	1	1	Mm1
....cacaguuugaGuccagggc...................	2	1	Mm1
....cacaguuugauuccaggAc...................	6	1	Mm1
....cacagCuugauuccagggc...................	1	1	Mm1
....cacaguuugauuccagUgc...................	1	1	Mm1
....cacaguuugauuccagggc...................	666	0	Mm1
....cacaguuugauuccagggU...................	1	1	Mm1
....cacaguuugauuccaggCc...................	3	1	Mm1
....cacaguuugauuccagAgc...................	3	1	Mm1
....cacaguuugaAuccagggc...................	2	1	Mm1

Star **Mature**

gaugauggaacaaccacagucgugguuuccgccccuguaaucacugagccaacugcagaacuggaugacaauuggcacaguuugauuccagggcugaaauccacaucaaccua			
....cacaguuugaCuccagggc...................	1	1	Mm1
....cacaguuugauuccagggcu..................	41	0	Mm1
....acaguuugauuccagggc...................	86	0	Mm1
....acaguuugauuccaggCc...................	1	1	Mm1
....acaguuugauuccagggA...................	1	1	Mm1
....caguuugauuccagggcA..................	5	1	Mm1
....caguuugauuccagggcG..................	2	1	Mm1
....caguuugauuccagggcu..................	66	0	Mm1
....caguuugauuccagggcuU.................	3	1	Mm1
.................................ccuguaaucacugagccaacu...	6	0	Mm2
.................................ccuguaaucacugagccaacug..	50	0	Mm2
.................................ccuguaaucacugagccaacGg..	1	1	Mm2
....cagaacuggaugacaauuggcaca..................................	2	0	Mm2
....Cauuggcacaguuugauuccagg.....................	1	1	Mm2
....Cauuggcacaguuugauuccagggc...................	3	1	Mm2
....auuggcacaguuugauucc........................	2	0	Mm2
....auuggcacaguuugauucca.......................	1	0	Mm2
....auuggcacaguuugauuccagg.....................	19	0	Mm2
....auuggcacaguuugauuGcagg.....................	1	1	Mm2
....auuggcacaguuugauGccaggg....................	1	1	Mm2
....auuggcacaguuugauuAcaggg....................	3	1	Mm2
....auuggcacaguuugauAccaggg....................	1	1	Mm2
....auuggcacaguuugauuUcaggg....................	1	1	Mm2
....auuggcacaguuugauuccaggA....................	1	1	Mm2
....auuggcacaguuugauuGcaggg....................	4	1	Mm2
....auuggcacagCuugauuccaggg....................	1	1	Mm2
....auuggcacaguuugauCccaggg....................	3	1	Mm2
....auuggcacagGuugauuccaggg....................	1	1	Mm2
....auuggcacaguuugauuccaggg....................	525	0	Mm2
....auuggcacaguuugauuccaggU....................	1	1	Mm2
....auuggcacUguuugauuccaggg....................	1	1	Mm2
....auuggcacagAuugauuccaggg....................	3	1	Mm2
....auuggcacaguuugGuuccaggg....................	1	1	Mm2
....auuggcacaguuugauuccaggC....................	4	1	Mm2
....auuggcacaguuCgauuccagggc...................	1	1	Mm2
....auuggcacaguuuUauuccagggc...................	3	1	Mm2
....Cuuggcacaguuugauuccagggc...................	1	1	Mm2
....auuggcacGguuugauuccagggc...................	1	1	Mm2
....auuggcacaguuugauuccagggG...................	1	1	Mm2
....auuggcacaguuugauuccagggc...................	10396	0	Mm2
....auuggcacaguuugauucUagggc...................	6	1	Mm2
....auuggcacaguuugauuccagggU...................	72	1	Mm2
....auuggcacaguuugauuccaAggc...................	3	1	Mm2
....auuggcCcaguuugauuccagggc...................	1	1	Mm2
....auuggcacaguuGgauuccagggc...................	1	1	Mm2
....auuggcacaUuuugauuccagggc...................	4	1	Mm2
....auuggcacaguuugauAccagggc...................	29	1	Mm2
....aCuggcacaguuugauuccagggc...................	3	1	Mm2
....auuggcacaguuugauGccagggc...................	18	1	Mm2
....auuggcacaguuugUuuccagggc...................	2	1	Mm2
....auuggAacaguuugauuccagggc...................	3	1	Mm2
....auuggcacaguuugauuAcagggc...................	76	1	Mm2
....auuggcacagCuugauuccagggc...................	16	1	Mm2
....auAggcacaguuugauuccagggc...................	2	1	Mm2
....auuggcacaguuugauCccagggc...................	28	1	Mm2
....auuggcacagGuugauuccagggc...................	12	1	Mm2
....auuggUacaguuugauuccagggc...................	2	1	Mm2
....auuCgcacaguuugauuccagggc...................	2	1	Mm2
....auuggcacaguuugaAuccagggc...................	2	1	Mm2
....auuggcacUguuugauuccagggc...................	14	1	Mm2
....auuggcacaguuugauuccaggAc...................	2	1	Mm2
....auugUcacaguuugauuccagggc...................	2	1	Mm2
....aAuggcacaguuugauuccagggc...................	8	1	Mm2
....auuggcacaguuugauuUcagggc...................	56	1	Mm2
....auuggcacaguuugauuccaggCc...................	3	1	Mm2
....auuggcacaguuugauucAagggc...................	1	1	Mm2
....auuggcacaguuugauuGcagggc...................	48	1	Mm2
....auuggNacaguuugauuccagggc...................	2	1	Mm2
....Nuuggcacaguuugauuccagggc...................	2	1	Mm2

```
                                     Star                                     Mature
gaugauggaacaaccacagucgugguuuccgccccuguaaucacugagccaacugcagaacuggaugacaauuggcacaguuugauuccagggcugaaauccacaucaaccua
......................................................................auuggcacaguAugauuccagggc...................   1    1   Mm2
......................................................................auuggcacaguuugaCuccagggc...................   2    1   Mm2
......................................................................auuggcacagAuugauuccagggc...................   21   1   Mm2
......................................................................auuggcacaguuugauuccaggUc...................   2    1   Mm2
......................................................................auuggcNcaguuugauuccagggc...................   1    1   Mm2
......................................................................auuggcacaguuuCauuccagggc...................   1    1   Mm2
......................................................................auuggcacaAuuugauuccagggc...................   1    1   Mm2
......................................................................auuAgcacaguuugauuccagggc...................   1    1   Mm2
......................................................................auuggcGcaguuugauuccagggc...................   13   1   Mm2
......................................................................auuggcaUaguuugauuccagggc...................   2    1   Mm2
......................................................................auuggcacaguuugauuccagggA...................   37   1   Mm2
......................................................................auGggcacaguuugauuccagggc...................   3    1   Mm2
......................................................................auuUgcacaguuugauuccagggc...................   1    1   Mm2
......................................................................Guuggcacaguuugauuccagggc...................   10   1   Mm2
......................................................................auugAcacaguuugauuccagggc...................   2    1   Mm2
......................................................................Uuuggcacaguuugauuccagggc...................   11   1   Mm2
......................................................................auuggcacaguuugauuccagggcG..................   5    1   Mm2
......................................................................auuggcacaguuugauuccagggUu..................   4    1   Mm2
......................................................................auuggcacaguuugauuGcagggcu..................   2    1   Mm2
......................................................................auuggcacaguuugauuccagggAu..................   3    1   Mm2
......................................................................auCggcacaguuugauuccagggcu..................   1    1   Mm2
......................................................................auuggcacaguuugauuccagggcC..................   1    1   Mm2
......................................................................auuggcacaguuugauAccagggcu..................   2    1   Mm2
......................................................................auuggcacaguuugauuAcagggcu..................   2    1   Mm2
......................................................................auuggcacaguuugauuccagggcu..................   244  0   Mm2
......................................................................auuggcacaguuugauuccagggcA..................   6    1   Mm2
......................................................................auuggcacaguuugauuccagggcug.................   3    0   Mm2
......................................................................auuggcacaguuugauuccagggcuA.................   1    1   Mm2
......................................................................auuggcacaguuugauuccagggcuU.................   13   1   Mm2
.......................................................................uuggcacaguuugauuccagggc...................   8    0   Mm2
........................................................................uggcacaguuugauuccagggc...................   3    0   Mm2
.........................................................................ggcacaguuugauuccagggc...................   3    0   Mm2
..........................................................................Acacaguuugauuccaggg....................   1    1   Mm2
..........................................................................gcacaguuugauuccagggc...................   8    0   Mm2
..........................................................................Acacaguuugauuccagggc...................   1    1   Mm2
..........................................................................gcacaguuugauuccagUgc...................   1    1   Mm2
...........................................................................cacaguuugauuccagggc...................   116  0   Mm2
...........................................................................cacaguuugauuccaggUc...................   3    1   Mm2
...........................................................................cacaguuugauuccaggCc...................   2    1   Mm2
...........................................................................cacaguuugauuccagggcu..................   17   0   Mm2
...........................................................................cacaguuugauuccagggcG..................   1    1   Mm2
...........................................................................cacaguuugauuccagggcuU.................   1    1   Mm2
............................................................................acaguuugauuccagggc...................   20   0   Mm2
............................................................................acaguuugauuccagggU...................   1    1   Mm2
............................................................................acaguuugauuccagggcu..................   2    0   Mm2
.............................................................................caguuugauuccagggcG..................   2    1   Mm2
.............................................................................caguuugauuccagggcC..................   1    1   Mm2
.............................................................................caguuugauuccagggcA..................   1    1   Mm2
.............................................................................caguuugauuccagggcu..................   40   0   Mm2
.............................................................................caguuugauuccagggcug.................   1    0   Mm2
.............................................................................caguuugauuccagggcuU.................   4    1   Mm2
```
